# The Religious Meaning System and Resilience in Spouse Caregivers of Cancer Patients: A Moderated Mediation Model of Hope and Affect

**DOI:** 10.1007/s10943-021-01278-7

**Published:** 2021-05-26

**Authors:** Dariusz Krok, Beata Zarzycka, Ewa Telka

**Affiliations:** 1 Institute of Psychology, University of Opole, Plac Staszica 1, 45-052, Opole, Poland; 2 Institute of Psychology, John Paul II Catholic University of Lublin, Lubin, Poland; 3 The Maria Sklodowska-Curie Cancer Center, Institute of Oncology in Gliwice, Gliwice, Poland

**Keywords:** The religious meaning system, Resilience, Hope, Positive and negative affect, Spouse caregivers

## Abstract

The character of the relationship between religiosity and resilience depends to a large extent on mediation and moderation mechanisms which rely on cognitive and emotional processes. Research conducted within hope theory and the broaden and build theory indicates that hope and affect can mediate and moderate this relationship. The present study explored whether the relationship of the religious meaning system with resilience in spouse caregivers of cancer patients can be mediated by hope and simultaneously moderated by positive and negative affect. A total of 241 spouse caregivers completed a set of questionnaires. The results revealed that hope mediated the relationship between the religious meaning system and resilience. Furthermore, positive affect but not negative affect moderated the indirect effect of the religious meaning system to resilience through hope.

## Introduction

A large body of research has demonstrated the adverse consequences of everyday caregiving to cancer patients on the physical and mental health of their carers, as well as existential and spiritual consequences (Luckett et al., 2019; Zheng et al., 2021). Due to the complex physiological and psychological aspects of cancer, daily caregiving demands require significant attention and personal involvement, which can cause negative effects, e.g. fatigue, sleep difficulties, somatic complaints, hopelessness, or anxiety. Consequently, spouses of cancer patients tend to mobilise personal religious and non-religious resources that can strengthen their mental and physical capacities and offer a sense of purpose and meaning. Although research has clearly demonstrated the beneficial effects of religion and hope on well-being and mental adjustment among cancer patients and their caregivers (Jeter, 2016; Zarzycka et al., 2019), little is known about both the role of affect and underlying mediation and moderation effects in forming resilient attitudes.

### Religiosity, Hope, and Resilience

In the context of cancer, religious beliefs and practices can be a vital source of resilience as they offer personal strength in times of adversity (stress, chronic illness, trauma, tragic events). This is primarily due to the fact that individuals can derive meaning and purpose from religion, which, in turn, predisposes them to both perceive stressful life events in a more optimistic manner and mobilise their coping abilities (Faigin & Pargament, 2011; Vitorino et al., 2018). While facing challenges and burdens, people can find constructive solutions and explanations in the form of religious/spiritual support that enables them to overcome adverse events. It is therefore understandable that religiosity is associated with resilience which is usually understood as a personality characteristic that reflects one’s ability to effectively adjust and adapt to challenging life circumstances (Manning, 2013; Munoz et al., 2017). Resilience can also be defined as a dynamic process through which individuals are able to regain or preserve their mental health while being exposed to significant adversity (Moeller-Saxone et al., 2015). In this sense, resilience also appears to be particularly instrumental for cancer patients’ caregivers who are often overburdened with physical and emotional challenges.

Previous research has demonstrated clear connections between religiosity and resilience. Faigin and Pargament (2011) claimed that religion through spiritual coping strategies can provide constructive solutions to challenging and stressful life problems that positively influence resilience. Positive religious coping that consists of having secure relationships with God and experiencing feelings of spiritual connectedness with other people was found to be associated with greater well-being and better mental adjustment in both clinical and non-clinical groups (Pargament et al., 2013; Park et al., 2018). Thus, benevolent attitudes with the sacred and fellow believers can play a positive role in one’s resilience levels and successful adaptation to adversity. Nevertheless, religion can also cause distress to individuals in the form of religious/spiritual struggles; these are experiences of distress and conflict in the sphere of religion and spirituality and tend to result in negative mental outcomes. Studies have identified that even after controlling for socio-demographic and personality factors, religious/spiritual struggles were positively associated with depression and anxiety and negatively with life satisfaction and happiness (Abu-Raiya et al., 2015; Wilt et al., 2017). This form of religiosity may thus create distress and decrease adaptation to adversity.

The empirical evidence suggests positive associations between religion and resilience in caregivers working with different groups of patients. In a group of female family caregivers of people with severe disability, religious involvement was related to more optimal adaptation, with stronger associations among those who were older, spouses, and black (Koenig et al., 2016). Examining Alzheimer patients’ caregivers, Hemalatha and Banu (2018) revealed a significant positive relationship between religion and resilience; religious beliefs and behaviour seemed to facilitate affirmative and empathetic attitudes of caregivers through providing ‘emotional nourishment’. In a longitudinal study, spiritual interventions based on religion and spiritual lessons were able to reinforce resilience in parental caregiving of children with autism (Pandya, 2018). Similar results were also found in older adults for whom religious service attendance was linked to higher resilience (Manning & Miles, 2018). Examining informal family carers, Heath et al. (2018) concluded that religious and spiritual values seemed inherently influential during many decisions made by the carers.

Although a relationship between religiosity and resilience seems firmly established, little is known about psychological factors that can mediate this relationship. According to Snyder’s comprehensive theory of hope (Snyder, 1994; Snyder et al., 2002), one of the factors that can have significant mediating importance for both religiosity and resilience is hope. (Snyder et al., 1999; Snyder et al., 2003) argue that human actions are directed towards goals and, in this context, hope is a cognitive set of goal-directed expectations that comprise two major dimensions: hope agency and hope pathways. The first represents one’s cognitive appraisals of being able to achieve certain desired goals, while the latter embodies one’s cognitive appraisals of potential pathways to goals. Snyder also maintains that hope has the capacity to influence resilience due to the cognitive-motivational characteristics that are instrumental in achieving one’s desired goals and overcoming life adversities.

Describing the relationship between religion and hope, Snyder et al. (2002) pointed out that religion provides a coherent set of valuable goals related to religious and moral values, clear pathways for achieving those goals in the form of rules and laws, and agency thinking through which people can proceed along pathways to complete goals. Later research has confirmed relationships between religiosity and hope. Religiosity was connected to hope in women with breast cancer, as well as hope mediated associations between religiosity and coping styles (Hasson-Ohayon et al., 2009). In a group of family caregivers of ill patients, Plakas et al. (2011) demonstrated that religiosity was a significant factor which enhanced feelings of hope and provided strength for the caregivers to confront adverse situations.

Associations were also found between the religious meaning system, which is understood as a cognitive and motivational system reflecting the religiously oriented categories of significance and purpose, and hope among late adolescents (Krok, 2016b). Specifically, the religious meaning system was positively associated with both basic hope and hope for success, and, in addition, meaning in life mediated the relationship between the religious meaning system and hope for success. Based on a cognitive approach, the religious meaning system represents the orienting and meaning-making function of religion in regulating human cognitions, feelings, and behaviour (Krok, 2014, 2016a). It enables people to explain and understand both elements of the external world (e.g. metaphysical phenomena in the world, causal principles in nature) and particular events occurring in their lives (e.g. the multifaceted experience of suffering, the inevitability of death). The religious meaning system includes two dimensions: (1) orientation that enables individuals to orientate themselves and understand the world and their own lives and (2) meaningfulness that includes the perspective of interpreting life in terms of meaning and purpose.

The relationship between hope and resilience derives from the observation that both constructs are also closely associated with goal-directed activities undertaken in the context of adversity (Munoz et al., 2017). Research indicated that hope contributed to the subjective experience of resilience; these associations mainly occurred on a shared basis of goals that had a crucial role in initiating goal-directed actions in the context of difficulties and challenges (Kirmani et al., 2015; Munoz et al., 2017). Accentuating attainable goals of competence and concentrating on positive goals, rather than evading potential problems and traps may foster positive adaptation and growth in the context of high risk or adversity (Masten, 2013). It thus seems plausible to point to goals as the common ground for resilience and hope.

Both hope and resilience refer to a domain of cognitive-motivational capacities which enable individuals to cope with adverse situations and constructively adapt to any emerging obstacles. Examining mother caregivers of children suffering from chronic physical illnesses, Horton and Wallander (2001) revealed that hope was a resilience factor as it was negatively associated with caregiver-related distress. The salutary effect of hope was especially noticeable when mothers experienced a high level of stress caused by their caregiver duties and responsibilities. Bally et al. (2014) also established that hope was instrumental in building the resilience skills of parents caring for children with cancer because of its comforting and strengthening effects and the ability to offer inner guidance in times of distress and uncertainty.

There have been studies demonstrating that hope was a mediator between religiosity and psychological factors, some of which might be related to resilience. In a group of amyotrophic lateral sclerosis caregivers, Jeter (2016) proved that hope mediated the relationship of spirituality with psychological well-being. Examining university students and their family members, Nell and Rothmann (2018) revealed that hope mediated the association between religiosity and subjective well-being. Hope was also found to mediate relationships between religiosity and psychological characteristics that had adverse relationships with resilience, e.g. depression in primary care adults (Chang et al. 2013). Such findings indicate that hope is likely to play a mediational role in the relationship between religiosity conceptualised as the religious meaning system and resilience largely on the basis of goals as it can enable individuals to formulate and accomplish their religious, moral, and ethical goals, and strengthen their goal-directed activities, which, in turn, is beneficial in overcoming adversities and facilitating successful adaptation.

### The Moderating Role of Affect

Although there have not been direct studies demonstrating the moderating role of affect in relationships among religiosity, hope, and resilience, previous research conducted on caregivers emphasised an important function of affect in the domains of goal-directed and resilient behaviour. Positive emotions experienced by family members caring for terminal cancer patients were a significant source of the caregiver’s self-worth and perseverance in the face of distress and challenges (Grbich et al., 2001). Religious service attendance, positive emotions, and hope for a better future were beneficial in helping family caregivers of people with mental illness to cope with the sustained stress of caregiving and to overcome adversities (Chadda, 2014). Different dimensions of religiosity (e.g. importance of religion, religious attendance) were related to lower depression among caregivers of people with dementia (Winter et al., 2015). Religiosity was also positively associated with hope and positive affect, but not so with negative affect; in contrast, hope was positively associated with positive affect and negatively associated with negative affect in a group of university students (Fadardi & Azadi, 2017). Considering that positive affect may be linked to one’s ability to find constructive pathways for accomplishing goals, caregivers may be more likely to engage in goal-directed activities leading to overcoming adversities and adaptation in the context of caring for their relatives.

As regards resilience, negative emotional states, i.e. distress and anxiety, were conversely correlated with psychosocial resiliency factors in caregivers of intensive care patients (Shaffer et al., 2016). Examining caregivers of advanced cancer patients, Palacio et al. (2018) revealed through regression analyses that emotional distress was negatively associated with caregivers’ resilience. Those relations can be understood within the broaden and build theory (Fredrickson, 2001), which assumes that positive emotions contribute to well-being by broadening perception, thoughts, and actions. Drawing on the theory, Tugade et al. (2004) suggested that experiences of positive emotion were conducive to building resilience in the form of being able to effectively ‘bounce back’ from stressful experiences. Taken together, it seems likely that two forms of affect, namely positive and negative, can moderate the association between religiosity and hope. We thus expected positive and negative affect to exert different effects on the relationship between religiosity and hope.

### The Present Study

**Fig. 1:**
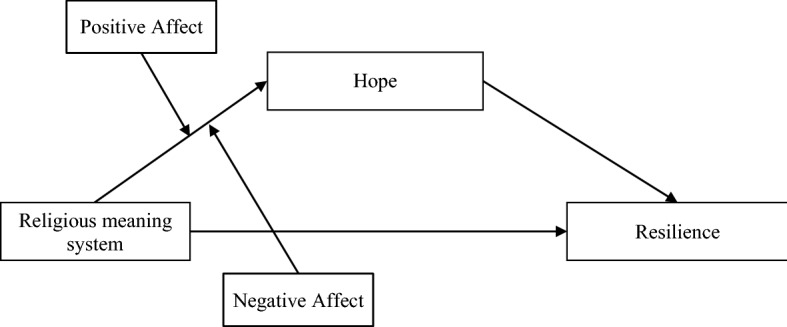
The general moderated mediation model

This study tested an integrated moderated mediation model which aimed to examine the relationship between the religious meaning system and resilience in spouses of cancer patients (Fig. 1). To date, the hypothesis that affect moderates the mediating relationship among the religious meaning system, hope, and resilience has not been tested. Based on both the broaden and build theory and previous research, the following hypotheses were proposed: H1: Hope mediates the relationship between the religious meaning system and resilience; H2: Positive affect moderates the indirect effect between the religious meaning system and resilience through hope; specifically, the positive association between the religious meaning system and hope is stronger when positive affect is high vs low; H3: Negative affect moderates the indirect effect between the religious meaning system and resilience through hope; specifically, the positive association between the religious meaning system and hope is weaker when negative affect is high vs low.

The sample size was determined on a basis of a priori power analysis related to moderated mediation models, as recommended by Preacher et al. (2007). The analysis specified a sample size of *n* = 100 or more persons as necessary to estimate a statistical power of over 0.80 in all variables (*p* = 0.05). Furthermore, mediation and moderation mediation analyses were conducted by using bootstrapped samples = 5000 (95% bias-corrected confidence intervals), so the sample in our study (*n* = 100 or more) prevents us from Type II error rates.

## Method

### Participants

Two hundred and fourteen spouses of gastrointestinal cancer patients were recruited in either hospital units or during scheduled appointments at outpatient medical centres. Criteria for exclusion were inability to fill in the questionnaires due to medical reasons (e.g. illness, poor eyesight, or physical weakness), cognitive deficiencies (e.g. serious problems with memory or another mental function, medical history on psychiatric disorders, Alzheimer’s disease or another type of dementia, or inability to fulfil daily activities requiring normal mental functions), the spouse being in the palliative phase (grade 4 according to clinical and diagnostic criteria), or sporadic contact with the spouse (i.e. the spouse only contacted the sick person once a week maximum or was absent from home for personal/work reasons for more than 3/4 of the total time). Two physicians were consulted to assist in identifying and reporting the first three medical conditions. Criteria for inclusion were more than 18 years of age, being with the spouse for more than 1 year, and ability to fill in the questionnaires. Two hundred and fifty-one spouses were initially recruited to participate in our study; thirteen spouses refused to participate and twenty-four did not return the questionnaires. Consequently, the final sample consisted of two hundred and fourteen spouses (118 women and 96 men), who ranged in age from 27 to 83 years. The overall participation level was 85.25%. Participant demographic characteristics are given in Table 1.

**Table 1:** Participant demographic characteristics

Participants	*M*	SD
Age	51.16	12.08

	*N*	*%*
*Gender*		
Male	96	44.8
Female	118	55.2
*Work status*		
Active	134	62.6
Not active	80	37.4
*Religion*		
Catholic	172	80.4
Protestant	21	9.8
Atheist	11	5.1
Agnostic	10	4.7
*Education*		
Basic vocational education	46	21.4
High school education	105	49.2
University education	63	29.4
*Children*		
1 child	30	14.0
2 children	144	67.3
> 3 children	34	15.9
No children	6	2.8

### Procedure

Participants were approached personally while visiting their spouses in hospital or asked to fill in the questionnaires through their spouses at home. Research assistants presented it to the participants, explained all its details, and gave them the questionnaires in a closed envelope to be filled in within 2 weeks. Ethical and professional care was also available in the context of the participants’ vulnerability. After the study, the spouses were debriefed and provided with answers to their potential queries which were mainly related to the purpose of the study, the name and type of institution which carried out the study, and the potential use of the findings. Ethical approval for the study was granted by the Institutional Ethics Committee at the University of Opole.

### Measures

#### Religiosity

The Religious Meaning System Questionnaire (Krok, 2009, 2014) was used to assess religiosity. The questionnaire was developed to conceptualise religiosity as a cognitive and motivational system allowing people to comprehend and interpret their personal experiences in terms of orientation and meaning. It has two subscales: orientation and meaningfulness; the total score is calculated by adding together these scores. The questionnaire consists of 20 items which are rated on a seven-point scale, ranging from 1 (very strongly disagree) to 7 (very strongly agree). The Cronbach’s coefficient for the present study was 0.90 (total score).

#### Affect

The Positive and Negative Affect Schedule (PANAS-X; Watson & Clark, 1999) was used to evaluate positive and negative affect. The scale contains 60 items which allows researchers to measure positive and negative affect, as well as 11 more specific emotions. As our main purpose was to assess the moderating role of general affect, we only applied the positive and negative affect subscales. Each of these comprises 10 items which are rated on a five-point Likert scale, ranging from 1 (very slightly or not at all) to 5 (extremely). The Cronbach’s coefficients for the current study were 0.85 (positive affect) and 0.87 (negative affect).

#### Hope

The Questionnaire of Hope for Success (Łaguna et al., 2005) measures hope conceptualised as one’s belief that they possess sufficient abilities to achieve success and their desired goals. It consists of 12 items, of which 8 are diagnostic, rated on an eight-point Likert scale ranging from 1 (definitely untrue) to 8 (definitely true). The questionnaire includes two subscales: the ability to find solutions and willpower; their sum provides the total score. The Cronbach’s coefficient for the present study was 0.86 (total score).

#### Resilience

The Resiliency Assessment Scale (Oginska-Bulik & Juczynski, 2008) evaluates resilience as a personality characteristic that helps individuals cope with adversities. The scale consists of 25 items rated on a five-point Likert scale, ranging from 0 (definitely not) to 4 (definitely yes). It has five subscales: (a) determination and persistence in activities, (b) competencies in coping and overcoming negative affective states, (c) openness to new experiences and humour, (d) tolerance of failures and challenges, and (e) optimistic life outlook. A total score is computed by adding their results. Taking into account relatively high correlations between the subscales and the total result, we decided to use only the total score as the results for particular subscales would be almost identical. The Cronbach’s coefficient for the present study was 0.81.

## Results

### Descriptive Statistics and Correlations among the Religious Meaning System, Hope, Affect, and Resilience

First, we examined the correlations among the religious meaning system, hope, affect, and resilience; these are illustrated in Table 2.

**Table 2:** Descriptive statistics and correlations among the religious meaning system, hope, affect, and resilience

Variables	*M*	*SD*	1	2	3	4	5
1. Religious meaning system	4.19	1.14	‒				
2. Hope	5.25	0.85	0.18**	‒			
3. Positive affect	3.45	0.61	0.08	0.23***	‒		
4. Negative affect	2.03	0.71	0.03	− 0.21**	− 0.31**	‒	
5. Resilience	5.08	0.88	0.15*	0.37***	0.66***	− 0.55***	‒

***p < 0.001; **p < 0.01; *p < 0.05

Most of the correlations between the variables were statistically significant. Nevertheless, the correlation coefficients had different signs depending on particular variables. The religious meaning system was positively related to hope and resilience. Hope, meanwhile, was positively associated with positive affect and resilience but negatively associated with negative affect. Finally, resilience was positively related to positive affect and negatively related to negative affect. The results of these correlational analyses enabled us to examine a mediation model: the religious meaning system‒hope‒resilience.

### Mediation Analysis

In the next step of the statistical analyses, we conducted a mediation analysis (Model 4 was applied) with the bootstrapping procedure to investigate the direct and indirect effects (samples = 5000; 95% bias-corrected confidence intervals) (Hayes, 2018). The main aim was to assess whether hope would mediate the relationship between the religious meaning system and resilience (Table 3).

**Table 3:** Mediation estimates for hope in mediating the relationship between the religious meaning system and resilience

Variables	*B*	SE	*t* [LLCI, ULCI]	Model *R*^2^
*Direct effects*				
Religious meaning system–Hope	0.07	0.03	2.66 [0.02, 0.12]	0.04**
Hope–Resilience	0.59	0.10	5.51 [0.38, 0.80]	–
Religious meaning system–Resilience	0.06	0.04	1.34 [− 0.03, 0.14]	0.15***
*Total effect*				
Religious meaning system‒Resilience	0.10	0.04	2.22 [0.01, 0.19]	–

Indirect effect	Effect	SE	LLCI	ULCI
Religious meaning system–Hope‒Resilience	0.04	0.02	0.01	0.09
Ratio of indirect to total effect of Religious meaning system on Resilience	0.43	8.54	0.08	2.26

***p < 0.001, **p < 0.01

The results of direct effects demonstrated that the religious meaning system was positively associated with hope. In addition, hope was positively associated with resilience. However, the direct effect between the religious meaning system and resilience was statistically non-significant. As regards indirect effects, hope turned out to mediate the relationship between the religious meaning system and resilience. The religious meaning system was related to higher hope, which in turn was related to a higher level of resilience. The direct effect of the religious meaning system on resilience was non-significant, which technically indicates mediation. It is also confirmed by the significant ratio of indirect to total effect of religious meaning system on resilience. Hypothesis 1 was thus fully confirmed.

### Moderated Mediation Analysis

The final step of the statistical analyses is centred around evaluating the moderating function of positive and negative affect on the indirect effects between the religious meaning system and resilience through hope. A moderated mediation analysis with the bootstrap procedures (samples = 5000; 95% bias-corrected confidence intervals) was performed to examine the potential associations (Model 9 was applied) (Hayes, 2018). The results are presented in Table 4.

**Table 4:** Moderated mediation estimates for resilience outcomes

Variables	*B*	SE	*t*[LLCI, ULCI]	Model *R*^2^
*Direct effects*				
Religious meaning system–Hope	0.07	0.03	2.43 [0.02, 0.11]	–
Positive affect–Hope	0.16	0.03	2.70 [0.04, 0.28]	–
Negative affect–Hope	− 0.11	0.05	− 2.15 [− 0.21, − 0.01]	–
Interaction: Religious meaning system x Positive affect	0.09	0.03	2.40 [0.01, 0.16]	–
Interaction: Religious meaning system x Negative affect	− 0.02	0.04	− 0.40 [− 0.10, 0.07]	0.14***
Hope‒Resilience	0.59	0.10	5.51 [0.38, 0.80]	
Religious meaning system‒Resilience	0.06	0.04	1.34 [− 0.03, 0.14]	15***

Conditional indirect effects	Effect	SE	LLCI	ULCI
Low positive affect x low negative affect	0.01	0.03	− 0.06	0.07
Low positive affect x high negative affect	− 0.01	0.02	− 0.05	0.04
High positive affect x low negative affect	0.08	0.03	0.02	0.15
High positive affect x high negative affect	0.07	0.03	0.02	0.15
*Index of moderated mediation*				
Hope as a mediator and positive affect as a moderator	0.06	0.02	0.01	0.11
Hope as a mediator and negative affect as a moderator	− 0.01	0.03	− 0.07	0.05

***p < 0.001, **p < 0.01, *p < 0.05

There were significant direct effects between the religious meaning system and hope, positive affect and hope, and negative affect and hope. The first interaction between the religious meaning system and positive affect was significant. The index of moderated mediation was also significant for positive affect (indirect effect = 0.06, CI_95_ = 0.01, 0.11). The conditional indirect effect for spouses of cancer patients with high positive affect was stronger than that for spouses with low positive affect, which allowed us to confirm Hypothesis 2. However, the second interaction between the religious meaning system and negative affect turned out to be non-significant. The index of moderated mediation for negative affect was also non-significant (indirect effect = −0.01, CI_95_ = −0.07, 0.05), which further indicates that negative affect was not a moderator of the indirect effect of the religious meaning system on resilience through hope. These findings did not confirm Hypothesis 3, which proposed such interactive effects. Comparison between the mediation and moderated mediation models showed 13% of the variance explained in resilience (Δ*R*^2^ = 0.13). It demonstrated the moderating value of positive and negative affect on the indirect effects between the religious meaning system and resilience through hope.

## Discussion

The present study aimed to investigate the mediating role of hope and the moderating function of positive and negative affect in the relationship between the religious meaning system and resilience in spouse caregivers of cancer patients. Its results provide new evidence that hope and affect may influence the way in which religiosity relates to one’s adaption to adversity.

### The Mediating Role of Hope

Consistent with the first hypothesis, hope was found to mediate the relationship between the religious meaning system and resilience. Specifically, the religious meaning system was related to higher hope, which in turn was related to a higher level of resilience. These findings support previous studies in which hope mediated the associations between spirituality and psychological well-being among amyotrophic lateral sclerosis caregivers (Jeter, 2016), in addition to between religiosity and subjective well-being in university students and their family members (Nell & Rothmann, 2018). Moreover, they also extend earlier research by illuminating the mediation effect played by hope. This assumes that positive adaptation to significant adversity among spouse caregivers is only present due to finding hope and that hope is associated with the form of religiosity that allows caregivers to comprehend and interpret their personal experiences through meaning systems. Due to the presence of goals in its structure, the religious meaning system provides individuals with the opportunity to understand the overall meaning of current events, which helps to strengthen hope and optimistic thinking.

The indirect route between the religious meaning system and resilience through hope suggests the occurrence of particularly interesting psychological mechanisms embedded in the domain of goals and purposeful action. Indeed, many authors have decisively noted that religion very rarely ‘operates’ on its own; in most cases, its effects are strongly interconnected with a broad spectrum of psychosocial factors which often mediate or moderate relationships between religious beliefs/behaviour and mental and physical health (Masters & Hooker, 2013; Morton et al., 2017; Park et al., 2018). One of the reasons why hope mediated the relationship of the religious meaning system with resilience among spouse caregivers can lie in the domain of goals and goal-directed activities which, to varying extents, is shared by the three factors.

The religious meaning system seems to be positively associated with hope by providing caregivers with a set of valued goals (e.g. inner harmony, unity with God, supernatural assistance in difficult times) and enhancing their ability to discern pathways leading to the accomplishment of desired goals. In addition, most religions prescribe a wide range of goals which have a strong motivational function that enables individuals to adapt to stressful situations, e.g. care-related challenges and demands. This view is supported by Schnitker and Emmons (2013), who showed that spiritual goals, i.e. goals connected to the sacred, influence cognition and behaviour by providing meaning and agency. The extant literature also demonstrated a single factor underlying both meaning and hope (Feldman & Snyder, 2005), which subsequently explains the concomitant ties between religiosity expressed by the religious meaning system and hope in spouse caregivers of cancer patients.

The caregivers who are characterised by a high level of hope are able to recognise different pathways as being available for their religious and secular goals. At the same time, the awareness of divine providence and support will strengthen their confidence that they can accomplish their desired goals and meet religious obligations. They are also convinced that they possess the capacity to produce constructive strategies needed in times of adversity and challenges. In this sense, religion is vital to formulating goals and motivating individuals to achieve them, which underpins hope (Krok, 2016b; Nell & Rothmann, 2018). This mechanism is very likely to reinforce the caregivers’ belief in their ability to successful adaptation and help them reinterpret negative events through ‘a spiritual lens’, ease the hardship of daily demands, and offer a sense of hope (Pargament et al., 2013; Vitorino et al., 2018).

### The Moderating Effects of Positive and Negative Affect

The main finding presents a moderated mediation effect showing an interaction between affect and hope, whereby both positive and negative affect were entered into the model. Its results demonstrated that the indirect effect of the religious meaning system to resilience through hope was contingent on positive affect experienced by spouse caregivers of cancer patients. The indirect effect for the caregivers characterised by high positive affect was stronger than for those with low positive affect, which confirmed Hypothesis 2. In contrast, negative affect did not turn out to be a moderator of the aforementioned indirect effect analyses, which did not allow the research to verify Hypothesis 3.

These findings are highly interesting as they reveal two important aspects of affective functioning in spouse caregivers of cancer patients. First, the role played by positive affect is far more significant than by negative affect. Positive emotions enhance the awareness of successful goal pursuits in caregivers and strengthen their motivation to accomplish those goals. This expands on Chadda’s (2014) research examining positive emotions and hope in family caregivers by proving that positive emotions contribute to the caregiver’s perceived capacity to discover and follow cognitive paths to desired goals. Spouse caregivers are exposed to stressful caregiving demands and challenges which deplete their mental resources and cause a range of negative emotional effects (Palacio et al., 2018; Shaffer et al., 2016). Therefore, it is not surprising that spouse caregivers of cancer patients who face evident adversity and distress rely on positive emotions in order to ease tensions, enhance coping abilities, and generate hope-oriented activities. Subsequently, the caregivers can more effectively adapt to life’s challenges and build resilience.

Second, the moderation mediation effect obtained in the current study is clear evidence that the relationship of the religious meaning system with resilience is contingent upon the interplay of both goal-directed and affective factors; it is concurrently mediated by hope and moderated by positive affect. Despite a significant direct association between the religious meaning system and hope, this association is stronger for caregivers with a high level of positive affect, resulting in higher hope and, consequently, stronger resilience. Not only does this finding confirm previous studies on relationships between hope and resilience (Bally et al., 2014; Munoz et al., 2017), it also provides new evidence for future research by demonstrating that positive affect will indirectly benefit the caregiver’s ability to set goals and constructively develop pathways to accomplish those goals in order to facilitate resilient attitudes and behaviour in spouse caregivers of cancer patients. Within the hope theory, positive emotions can arise in relation to the pursuit and attainment of desired goals (Snyder et al., 2003). Pursuing important goals provides individuals with meaning in life and values, which consequently leads to positive feelings of self-fulfilment and inner satisfaction. The process of goal attainment embedded in hope and resilience is thus frequently linked to affective reactions.

This interpretation finds support in the broaden and build theory (Fredrickson, 2001; Tugade et al., 2004) in which caregivers who experience positive emotions are likely to consolidate personal resources by generating more optimistic thinking and increasing their awareness of available problem-solving activities which are invariably conducive to personal growth and goal-directed behaviour. Subsequently, this can contribute to better adaption to adversity and higher resilience. In this sense, positive emotions in spouse caregivers tend to ‘offset’ the detrimental effects of negative emotions caused by care-related strains. This can occur because positive emotions are able to enhance resilience, meaning in life, and coping skills in spouse caregivers (Grbich et al., 2001). By eliciting positive sensory experiences, positive emotions can infuse one’s life with positive appraisals and feelings, which will balance negative emotional states related to healthcare demands.

### Limitations of the Study

The current study has several limitations that warrant further consideration. First, the study focused on spouse caregivers of cancer patients, which prevents it from generalisation to other caregiver populations (e.g. caregivers of people with mental illnesses or disabled children) due to the clinical picture of cancer. Second, information regarding spouses’ use of other services, such as social care, psychiatric, or family help, was not collected, which may have affected their resilience abilities. Although this was done in order to not violate the caregivers’ privacy, the information could have slightly modified our results. Third, the study had a cross-sectional design, which invalidates the possibility of drawing causal conclusions about relationships regarding the variables entered into our model. Future research to better examine the longitudinal impact of affect and hope on spouse caregivers’ resilience will help establish more adequate relations.

#### Conclusion

In summary, the present study convincingly confirmed the significance of examining relationships between religiosity and resilience in spouse caregivers of cancer patients within a moderation mediation model. It demonstrated that hope mediated the association between the religious meaning system and resilience, which highlighted the critical role of goals and goal-directed behaviour in the domain of adaptation to adversity. More interestingly, the findings revealed that positive affect, but not negative affect, was a moderator of the indirect relation between the religious meaning system and resilience through hope; the indirect relation was stronger under the condition of higher positive affect. Through testing the moderation mediation effects, this research sheds new light on the interplay of goal-oriented and affective mechanisms in caregivers’ mental flexibility and adaptation, which also provides practical implications for family caregivers.

## Data Availability

The data that support the findings of this study are openly available in the OSF HOME repository at https://osf.io/7r2zx/, reference number: Spouses of CP 2.
